# Paving the Road to Tumor Development and Spreading: Myeloid-Derived Suppressor Cells are Ruling the Fate

**DOI:** 10.3389/fimmu.2015.00523

**Published:** 2015-10-12

**Authors:** Yaron Meirow, Julia Kanterman, Michal Baniyash

**Affiliations:** ^1^The Lautenberg Center for General and Tumor Immunology, Israel-Canada Medical Research Institute, Faculty of Medicine, The Hebrew University, Jerusalem, Israel

**Keywords:** cancer, MDSC, inflammation, immunosuppression, immunotherapy, chemotherapy, immune monitoring, biomarkers

## Abstract

Cancer development is dependent on intrinsic cellular changes as well as inflammatory factors in the tumor macro and microenvironment. The inflammatory milieu nourishes the tumor and contributes to cancer progression. Numerous studies, including ours, have demonstrated that the tumor microenvironment is immunosuppressive, impairing the anticancer immune responses. Chronic inflammation was identified as the key process responsible for this immunosuppression via induction of immature myeloid-derived suppressor cells (MDSCs). Upon a prolonged immune response, MDSCs are polarized toward immunosuppressive cells meant to control the exacerbated immune response. In cancer, the chronic inflammatory response renders the MDSCs harmful. Polarized MDSCs suppress T-cells and natural killer cells, as well as antigen-presenting cells, abrogating the beneficial immune response. These changes in the immunological milieu could also lead to high frequency of mutations, enhanced cancer cell stemness, and angiogenesis, directly supporting tumor initiation, growth, and spreading. The presence of MDSCs in cancer poses a serious obstacle in a variety of immune-based therapies, which rely on the stimulation of antitumor immune responses. Cumulative data, including our own, suggest that the selection of an appropriate and effective anticancer therapy must take into consideration the host’s immune status as well as tumor-related parameters. Merging biomarkers for immune monitoring into the traditional patient’s categorization and follow-up can provide new predictive and diagnostic tools to the clinical practice. Chronic inflammation and MDSCs could serve as novel targets for therapeutic interventions, which can be combined with conventional cancer treatments such as chemotherapy, radiotherapy, and cancer cell-targeted and immune-based therapies. Intervention in environmental and tumor-specific inflammatory mechanisms will allow better clinical management of cancer toward more efficient treatment.

## Introduction

While some cancers are triggered by intrinsic cellular changes such as germ-line mutations, the majority of tumors are caused by acquired somatic mutations due to alterations in homeostatic environmental factors. In many cases, tumor development is associated with nutritional and environmental factors, generated by chronically occurring pathologies such as infections, autoimmune disorders, metabolic diseases, and more. The common denominator of these processes is chronic inflammation, which is an abnormal and sustained form of a protective response. It starts as an acute inflammation; a beneficial response resolving insults such as newly encountered pathogens or transformed cells. However, when the stimuli that induce the immune response could not be cleared, a continued inflammatory response evolves. The prolonged response leads to loss of tissue homeostasis, resulting in a condition similar to unhealed wounds (1). This type of dysregulated responses occur at higher rates in cases of cancer, where transformed cells in conjunction with proinflammatory cells and factors result in tumor development and progression (2). On the one hand, growing tumors intensify the proinflammatory conditions that directly nourish their own growth and spreading. On the other hand, the prolonged response also creates an immunosuppressive environment by recruiting an array of suppressor cells and factors that enable escape of the tumor from immune surveillance (3).

In this review, we highlight the regulatory checkpoints controlled by immunosuppressive cells and factors in the tumor micro and macro environments, focusing on MDSCs: immunosuppressive cells which play a critical role in maintaining normal homeostasis and when polarized under chronic inflammatory conditions could skew the environment toward supporting tumor development and spreading.

## Immune System Homeostasis

Immune homeostasis is the body’s tightly regulated network of physiological checkpoints and balances that enables controlled defensive responses against pathogens or altered self-cells. In healthy individuals, an immune challenge by foreign or self-created insults initiates a cascade of appropriate responses that restore the tissue to its steady state. Homeostasis is transiently imbalanced when an acute inflammatory response is initiated to counteract a danger signal. In this regard, the acute inflammation is beneficial, as innate and adaptive immune cells are recruited and activated as long as the insult exists. Once the insult has been removed, the response is resolved and homeostasis is reconstituted, while enriching the host with a reservoir of memory and regulatory cells. These immune response cycles depend on a subtle balance between the effector and regulatory arms of the immune system and their capacity to clear the initial stimulus. The intricate cross talk between immune cells and soluble factors (cytokines and chemokines) ensues execution of pro- and anti-inflammatory responses at the appropriate time and location, thus preventing excessive and harmful immune activation. When the immune homeostasis is continuously disrupted, the host’s immune response can become hypo- or hyperactive. A compromised immune response poses severe immune deficiency-based complications, such as opportunistic infections and cancer. In contrast, autoimmune disorders are on the overactive side of the homeostatic spectrum. Thus, the maintenance of fine-tuned regulatory and effector immune functions is at the forefront of a balanced immune homeostasis.

Since excessive immune responses can be deleterious, regulatory mechanisms have evolved to suppress and resolve immune responses. There are several immunosuppressive cell populations, which regulate immune homeostasis by restraining aberrant or excessive immune responses. CD4^+^CD25^high^Foxp3^+^ regulatory T cells (Tregs) are essential for the maintenance of immune homeostasis, as their dysfunction leads to the appearance of widespread, fatal, immune-mediated diseases (4). Regulatory dendritic cells (rDCs) have the ability to regulate or inhibit T-cell activation and induce/promote Treg development and expansion (5, 6). Immature DCs induce tolerance due to inefficient antigen presentation and lack of costimulatory molecules (5, 7, 8). M2 macrophages play a role in wound healing by producing growth and angiogenic factors. However, they also have immunoregulatory activities as they produce regulatory cytokines (TNF-α, IL-1, IL-6, IL-10, and TGF-β) and overexpress arginase-1 (ARG-1) (9–12).

Cumulative evidence indicates that the most “powerful players” in turning off the immune response are immature myeloid cells (IMCs), also termed non-polarized or “resting” myeloid-derived suppressor cells (MDSCs). These cells were demonstrated to be tolerogenic and capable of suppressing immune system activities. In healthy individuals, these immature myeloid progenitors are retained in the bone marrow (BM) where they maintain a suppressive environment (13), most likely for preserving BM homeostatic niches. MDSCs display the capacity to migrate to the periphery and differentiate into mature macrophages, DCs, and neutrophils while losing their suppressive phenotype. Mouse MDSCs are characterized by CD11b^+^Gr1^+^ and can be subdivided into monocytic (CD11b^+^Ly6G^-^Ly6C^high^), which lack DC and macrophage markers (CD11c^−^, F4/80^−^, respectively) (14) and granulocytic (CD11b^+^Ly6G^+^Ly6C^low^) cells. The identity of human MDSCs is more intricate and based on several markers (CD11b^+^CD33^+^HLA-DR^−^), which could be further divided into monocytic (CD14^+^CD11b^+^CD33^+^HLA-DR^−^) and granulocytic (CD15^+^CD11b^+^CD33^+^HLA-DR^−^) phenotypes. Both granulocytic and monocytic subpopulations have been shown to be suppressive, especially the latter. However, the majority of MDSCs are granulocytic, making up in numbers for their milder suppressive ability (15). Under normal conditions, about 50–60% of the resident cells in mouse BM are resting Gr1^+^CD11b^+^ MDSCs, which exhibit a basal suppressive activity. Interestingly, mature T-cells that are occasionally circulating through the normal BM become reversibly dysfunctional, similar to peripheral T-cells during chronic inflammation (13). Under normal steady-state conditions in mice, MDSCs are also found in the spleen (3–5%) and in the peripheral blood (~10%) (3), displaying a basal immunosuppressive activity as well, most likely controlling homeostasis and resolving acute immune responses.

## Disrupted Immune Homeostatic Environment by a Developing Chronic Inflammation

### Chronic Inflammation Induces MDSC Polarization and Accumulation

Chronic inflammation develops when pathogenic or self-aberrant antigenic insults (e.g., cancer cells) are not resolved. The inflammatory immune response proceeds, albeit failing to eliminate the antigenic stimuli. The generated conditions are characterized by an influx of proinflammatory cells of the innate and adaptive immune systems. Such cells secrete cytokines, chemokines, and soluble factors that eventually create micro and macro environments that support tumor initiation and development. In parallel, the generated chronic inflammatory environment constantly recruits the regulatory arm of the immune system to resolve and control the inflammatory response. The continuous activity of the immune system together with the recruitment of inhibitory cells/factors eventually induces a general immunosuppression of T-cells and natural killer (NK) cells and in some cases of DCs. These indirectly support the tumor by enabling its escape from the immune effector functions (3).

The immunosuppressive conditions generated in the course of chronic inflammation begin with the induction of MDSC polarization: turning the cells into highly suppressive MDSCs, while their differentiation is arrested, maintaining them in an immature state. Polarized MDSCs expand in the BM, accumulate in the periphery, and invade the local inflammatory sites, where they confer a systemic and site-specific immunosuppression. It is not yet fully clear how exactly resting MDSCs convert into harmful immunosuppressive MDSCs. However, it is well established that under pathological conditions, increasing accumulation of growth factors [GM-CSF and VEGF (16)], chemokines [CXCL12 (17) and CCL2 (16)], and cytokines [TNF-α, IFN-γ, IL-1β, IL-6, and TGF-β (18)] accelerate MDSC expansion in the BM and accumulation in the periphery. Polarized MDSCs reach the blood stream, spleen, and sites of inflammation, as evident in various chronic pathologies such as inflammatory bowel diseases (IBD) and various types of cancer. They accumulate as well during bacterial infections such as *Helicobacter pylori* (19), *Salmonella enterica* serovar Typhimurium (20), and *Staphylococcus aureus* (21) and in parasitic infections such as *Schistosoma japonicum* (22). Interestingly, MDSCs do not enter peripheral lymph nodes, making them protected from the suppressive environment (13), unless metastases are evident in the lymph nodes (23).

**Figure 1:**
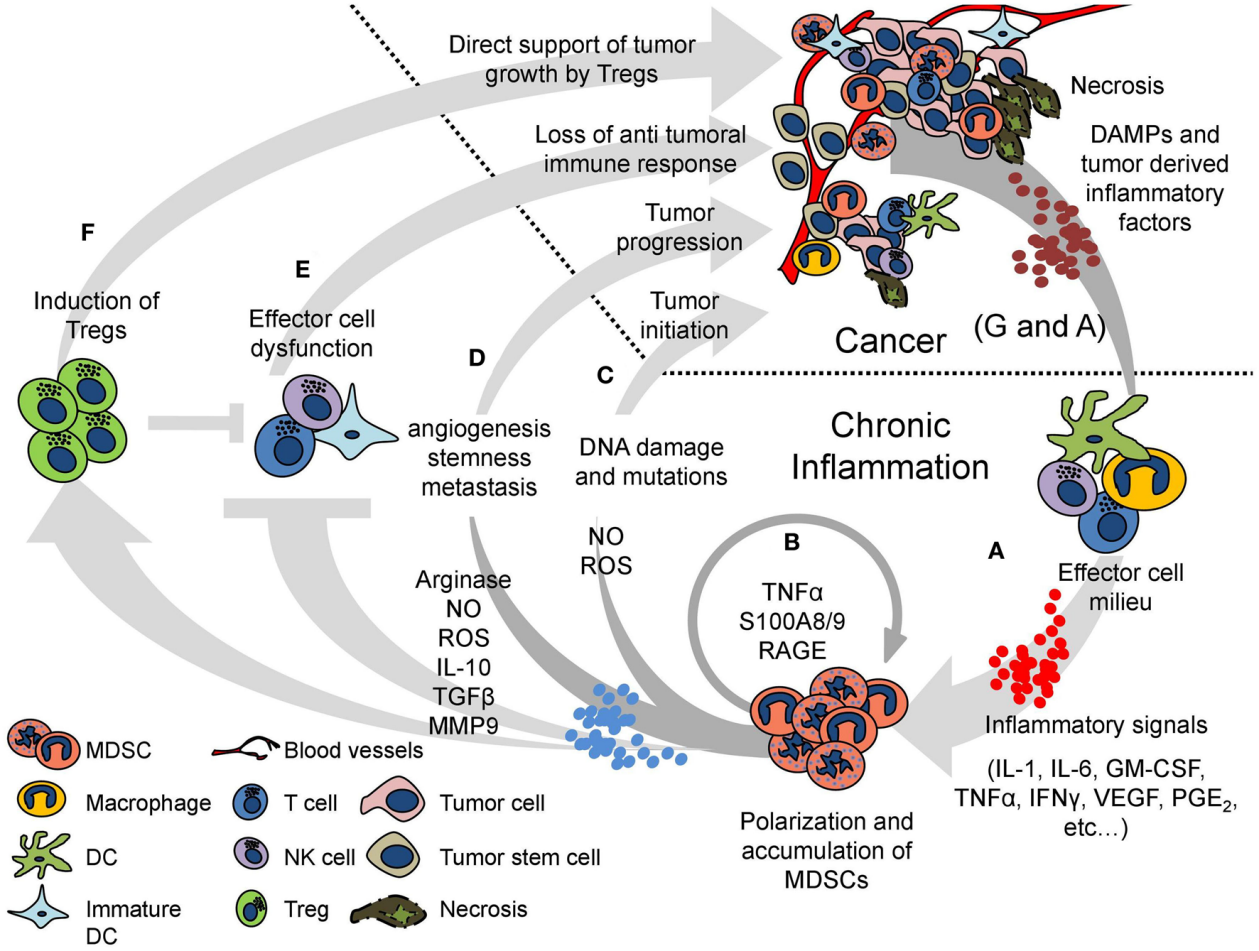
**A vicious cycle of chronic inflammation and cancer is maintained by MDSCs**. Cancer and chronic inflammation mutually support one another in multiple ways. The cycle begins with a chronic inflammatory response generated by any persistent insult, including cancer. The effector cell milieu is recruited and activated by DAMPs derived from the immunogenic insult and produces various proinflammatory signals (such as IL-1, IL-6, GM-CSF, TNF-α, IFNγ, VEGF, and PGE_2_) **(A)**. The proinflammatory signals persist and induce polarization and accumulation of MDSCs, which transform the beneficial inflammatory response to deleterious chronic inflammation. The MDSCs create a positive loop, increasing their own polarization and expansion by production of TNF-α and S100A8/9 proteins **(B)**. The oxidative stress created by the production of NO and ROS by the MDSCs in the inflammatory sites may cause DNA damage and somatic mutations, increasing the risk for tumor initiation **(C)**. MDSCs directly support tumor progression by increasing angiogenesis, tumor cell stemness, and metastasis **(D)**. The suppressive activity of MDSCs inhibits the effector function of T-, NK-, and dendritic cells, abrogating the antitumor beneficial response **(E)**. MDSCs further suppress the immune response by inducing Tregs, which can also directly support tumor growth by producing RANKL **(F)**. The deleterious inflammatory milieu and the tumor itself provide more proinflammatory factors, further enhancing MDSC accumulation. Moreover, necrosis at the tumor releases endogenous DAMPs, which could either start the cycle of chronic inflammation and MDSC accumulation or keep perpetuating it **(A,G)**.

We have recently shown that TNF-α plays a critical role in MDSC accumulation and suppressive function as it leads to myeloid cell differentiation arrest, which is accompanied by a specific polarization of these cells toward an immunosuppressive phenotype, inducing dysfunction of effector immune cells (18). TNF-α is generated by a variety of immune and non-immune cells such as T-cells, NK cells, macrophages and tumor cells, endothelial cells, mast cells, neurons, and fibroblasts (24). Interestingly, MDSCs themselves produce high levels of TNF-α, creating a positive autocrine and paracrine feedback that enhances their own polarization and accumulation. In addition to TNF-α, there is a variety of proinflammatory cytokines such as IL-1β, IL-6, and Type I and II interferons that are involved in establishing a proinflammatory environment. Such cytokines are produced upon recognition of pathogenic or endogenous ligands by Toll-like receptors (TLRs) expressed on various immune cells including MDSCs. Our group had previously described a mechanism in which persistent TLR activation by a single ligand was sufficient to induce MDSC accumulation and immunosuppression, similar to a pathology characterized by chronic inflammation. In this model, usually non-virulent danger-associated molecular patterns (DAMPs), such as LPS (TLR4), zymosan (TLR2), poly I:C (TLR3), or unmethylated CpG DNA (TLR9), induced a prolonged T_H_1 response accompanied by increased production of TNF-α and IFN-γ (25) (Figure 1A).

Myeloid-derived suppressor cell polarization is accompanied not only by their differentiation arrest but also by their enhanced immunosuppressive phenotype as reflected by the elevated activity of arginase-1 (ARG-1) and inducible nitric oxide synthase (iNOS), along with enhanced production of nitric oxide (NO) and reactive oxygen species (ROS). These effects are further enhanced by TNF-α (18, 26). The proinflammatory calcium binding S100 proteins, mainly S100A8/9, and their corresponding receptor, receptor for advanced glycation end products (RAGE), are upregulated in MDSCs in response to TNF-α. These proteins affect MDSCs in an autocrine and paracrine fashion to regulate MDSC polarization, accumulation, and differentiation arrest in a STAT-3-dependent mechanism (18, 27, 28) (Figure 1B).

### Immune System Dysregulation in the Course of Chronic Inflammation

The suppressive environment generated in the course of chronic inflammation is foremost generated by MDSCs. These cells were shown to directly abrogate the function of various effector cells of the innate and adaptive arms of the immune system while increasing the activity of regulatory mechanisms (Figures 1E,F).

#### T-Cell Dysfunction

We have demonstrated that chronically inflamed animals are more susceptible to viral infection due to dysfunction of the innate and adaptive immune systems. Mice with chronic inflammation were more sensitive to influenza infection associated with severely reduced survival rates (29). These results were further supported by the inability of both CD4^+^ and CD8^+^ T-cells to proliferate and produce IFN-γ in response to T-cell receptor (TCR) stimulation both *in vivo* and *in vitro* when cocultured with MDSCs (13, 18, 25, 29). MDSCs induce T-cell dysfunction by an array of mechanisms and perhaps the most prominent among these is the direct downregulation of CD247 [the ζ chain of the T-cell antigen receptor complex (TCR)]. CD247 is a 16 kDa transmembrane subunit of the TCR, containing within its long intracellular domain, three immune tyrosine based activation motifs (ITAMs), which make it the main signaling subunit of the TCR. CD247 is expressed in all T-cells and is crucial for TCR-mediated T-cell activation and serves as the limiting factor for the TCR complex expression on the cell surface. CD247 is easily detected both in mouse and human leukocytes with a specific antibody by Western blot or intracellular staining for flow cytometry (18, 29, 30). Under chronic inflammatory conditions, MDSCs induce lysosomal degradation of CD247 within the T-cells without affecting the surface levels of the remaining TCR subunits, leaving a functionally cryptic and inactive complex (29). This is a bystander effect causing dysfunction of all T-cells in an antigen non-specific manner. However, this phenomenon is reversible, since elimination of the chronic stimulus and/or MDSCs or neutralization of the inflammatory environment allows recovery of CD247 and T-cell function (13). The mechanisms applied by MDSCs to induce CD247 downregulation are still unknown.

l-arginine consumption by MDSCs is another key factor in the induction of T-cell dysfunction, which is governed by increased activity of ARG-1 and iNOS, using l-arginine as their substrate. l-arginine depravation was shown to cause the downregulation of CD247 in T-cells *in vitro* (31). A deeper mechanism has been described showing that l-arginine withdrawal from the environment leads to a G0–G1 cell cycle arrest in T-cells *in vitro* and a global reduction in protein synthesis in the cells (32). The enzymatic activity of iNOS is harmful to T-cell functions, as it generates NO that is responsible for suppressive consequences such as disrupting Jak3 and STAT5 activities (33), inhibition of MHC class II gene expression (34), and the induction of T-cell apoptosis (35). Nagaraj et al. showed that nitration of tyrosine residues of molecules involved in the immunological synapse is induced by MDSCs in an antigen-specific manner. The nitration correlated with the interference of TCR-CD8 complex assembly on the plasma membrane and a reduced antigen-specific TCR-mediated proliferation and IFN-γ production (36). In addition, MDSCs express galactin 9, which binds to the T-cell immunoglobulin domain and mucin domain 3 (TIM3), which is a negative regulator of T_H_1-cell responses on lymphocytes and induces T-cell apoptosis (37). MDSCs downregulate l-selectin (CD62L), a plasma membrane molecule necessary for the homing of naive T-cells to lymph nodes. This reduces the activation of CD4^+^ and CD8^+^ T-cells and inhibits homing to tumor draining lymph nodes (38).

#### NK-Cell Dysfunction

The suppressive environment generated by MDSCs in chronic inflammation leads to impaired NK-cell activity as well. Induction of chronic inflammation in mice prevents NK-cell-mediated allograft rejection *in vivo*, while elimination of MDSCs restores their ability to kill the allogeneic cells (18). CD247 is also expressed in NK cells, serving as a central signaling subunit of the NK killing receptors NKp46, NKp30, and FcγRIII (CD16), and is downregulated by MDSCs under chronic inflammatory conditions (25). Its downregulation may be a major reason for NK-cell dysfunction during chronic inflammatory conditions, including cancer settings. MDSCs also induce downregulation of the killing receptor NKG2D *in vivo* and *in vitro* (39). In addition to NKG2D downregulation, NK cells cocultured with MDSCs had reduced cytotoxic activity and IFN-γ production *in vitro*. These effects were shown to be cell contact dependent and were reversed by blocking membrane-bound TGF-β1 expressed on MDSCs, while soluble TGF-β did not suppress NK-cell activity (39). Blocking of NKp30 in cocultures of human MDSCs and NK cells was shown to reduce the suppressive effect of the MDSCs (40).

#### Prevention of Dendritic Cell Maturation

Accumulating data show that tumor residing DCs can retain an immature phenotype and induce tolerance to tumor antigens (41, 42). VEGF and IL-10 secreted in the tumor microenvironment constitutively activate STAT3 signaling in DCs, leading to downregulation of MHC class II molecules and costimulatory molecules, preventing DC maturation (43). Moreover, mature DC numbers inversely correlate with MDSC levels in cancer patients, suggesting that elevated MDSC levels, which are arrested at their immature stage, are prevented from differentiating to DCs (42). It is still unclear whether MDSCs can directly inhibit DC maturation and activity or the effect is a part of the skewed myelopoiesis in chronic inflammation. However, S100A8/9 proteins overexpressed in embryonic stem cells inhibited their ability to differentiate into mature DCs, abrogating their ability to induce T-cell proliferation *in vitro* (27). S100A8/9 proteins are highly expressed by MDSCs, suggesting a possible direct effect of MDSCs on DC differentiation via this pathway.

#### Induction of Regulatory T-Cells

In addition to inhibiting immune effector cells, MDSCs have been shown to activate regulatory cells to further induce immunosuppression. MDSCs were shown to induce expansion of Tregs by producing IL-10 and TGF-β (44). Tregs induced by antigen-loaded MDSCs caused induction of antigen-specific tolerance in a model of multiple myeloma. This mechanism, however, was independent of TGF-β (45).

## The Aggravating Consequences

### Chronic Inflammation Leads to Tumor Initiation and Cancer Development

Cancer is characterized by stepwise accumulation of genetic and epigenetic alterations of genes. As a high-risk factor for cancer, chronic inflammatory responses produce great amount of mediators, including cytokines, reactive oxygen and nitrogen species (ROS and NO, respectively), and proteinases, which can induce genetic and epigenetic changes of cancer-associated genes and pathways. An increased risk for cancer has been linked with various pathologies characterized by chronic inflammation such as IBD, papillomavirus, hepatitis C virus, and *H. pylori* infections that are linked to colon, cervix, liver, and gastric cancer, respectively (46–48). Chronic inflammation has also been thought to support progression of tumors, which were initiated by a non-immune carcinogenesis. Non-inflammatory tumors would be cleared by immune surveillance while inflammatory tumors would create chronic inflammation, induce MDSC accumulation, and abrogate the antitumor immune response (26, 28, 49).

The highly reactive oxidative agents ROS and NO, produced in the inflammatory settings, have been shown to cause DNA damage and induce somatic mutations (50). It was shown that in the case of IBD and *H. pylori* infection, the generated conditions lead to genomic instability in the mucosal layer and epithelial fractions of the colon and stomach, respectively, leading to tumor initiation (51, 52). This effect may be greatly enhanced when chronic inflammation develops and increasing numbers of NO/ROS producing MDSCs accumulate (Figure 1C). The skewed inflammatory environment generated by MDSCs leads to an uncontrolled cellular and factors milieu, ensuing in the establishment of an optimal niche for cancer initiation, tumor microenvironment enrichment, and support of a malignant progression. Furthermore, inflammation also modulates the expression of miRNAs and epigenetic changes that not only regulate the expression of tumor-related proteins but also enhance the tumor-promoting inflammation-associated carcinogenesis (53, 54).

Chronic inflammation with its associated cells, cytokines, chemokines, and soluble factors not only supports tumor initiation but also shapes the environment that nourishes the newly transformed cells as well as the progressive tumors to overgrow and invade. The effect could be direct, affecting the tumor cells or indirectly, by perpetuating an immunosuppressive environment. For example, MDSC levels were shown to correlate with serum VEGF levels in a mouse model of spontaneous mammary carcinoma (55). While MDSCs do not produce VEGF directly, they are affected by VEGF produced by almost all types of tumors (56). MMP9 produced by MDSCs has been suggested to augment VEGF production and enhance tumor vasculature, growth, and invasiveness in mouse models (57, 58). MDSCs have also been shown to increase metastasis by promoting formation of pre-metastatic niches and tumor cell invasion (59). Recently, MDSCs were shown to enhance the stemness of ovarian carcinoma cells, also increasing their metastatic capabilities, making the tumors more aggressive (60). Induction of Tregs by MDSCs in the settings of cancer and chronic inflammation provides another indirect mechanism that supports tumor progression, as tumor infiltrating Tregs have been shown to directly stimulate mammary cancer metastasis by interaction of receptor activator of NFκB (RANK) with RANK ligand on the Tregs (61). Apart from supporting tumor progression, cumulative data show that chronic inflammation itself may have continuous carcinogenic effects. A recent research has linked inflammation-induced transcription programs mediated by NFATc1-STAT3 complex to enhanced tumorigenicity in Kras G12D-mutant mice pancreatic tissue, while mutated Kras or an inflammatory response alone were insufficient to initiate cancer (62). Moreover, several inflammatory cytokines such as TNF-α, IL-6, TGF-β, and IL-10 have been shown to participate in both the initiation and progression of cancer. Proinflammatory cytokines such as TNF-α can induce DNA damage through ROS, which leads to tumor initiation. TGF-β can promote malignant transformation through epithelial–mesenchymal transition (EMT) activation. Cytokines derived from CD4^+^ lymphocytes, such as IFN-γ, IL-10, and IL-17, can participate in epithelial barrier disruption, M2 phenotypic transitions of macrophages, and angiogenesis, respectively. Tumor growth and invasion are also favored by proinflammatory cytokines that stimulate cell proliferation, reduce apoptosis, and enhance EMT and angiogenesis; the latter is facilitated by VEGF and IL-8. Anti-inflammatory cytokines, such as IL-10 and TGF-β, contribute to tumor immune evasion. Tumor-associated macrophages (TAM), tumor-infiltrating lymphocytes (TIL), and cancer-associated fibroblasts (CAF) secrete several factors that contribute to tumor growth and metastasis while maintaining the immunosuppressive milieu (63) (Figure 1D).

### Tumor Development Perpetuates Chronic Inflammation

Different tumors vary in their proinflammatory profile produced by both the tumor and the inflammatory milieu. In fact, some tumors start off as non-inflammatory, yet at a certain stage inflammation and MDSC accumulation is evident regardless.

Toll-like receptors and other pattern recognition receptors (PRRs) recognize endogenous danger signals as well as pathogen-associated molecular patterns. High-mobility group box protein 1 (HMGB1) and heat shock protein 60 (HSP60) have been shown to act as adjuvants activating APCs and inducing immune response (64–66). HMGB1 is a highly conserved vertebrate nuclear protein, and HSP60 is a highly conserved mitochondrial–cytosolic molecular chaperone. Both proteins are released to the extracellular matrix in necrotic tissue. Recently, HMGB1 and HSP60 have been shown to directly affect growth of murine mammary carcinoma *in vitro* in a MyD88-dependent mechanism (67), suggesting that a TLR recognition pathway is involved.

Necrosis in cancer, occurring due to insufficient vascularization and tissue damage in the tumor and its surroundings, results in accumulation of endogenous DAMPs in the tumor microenvironment. Similar to other TLR ligands, these DAMPs activate immune cells and induce a sustained T_H_1 response, leading to increased production of proinflammatory cytokines by tumor-infiltrating leukocytes. The generated inflammation persists as long as the tumor is present, perpetuating a vicious cycle of chronic inflammation, MDSC accumulation, and immunosuppression, which in turn drive disease progression (Figure 1G).

### Tumor-Derived Soluble Factors Drive MDSC Accumulation and Activation

The number and activity of the MDSCs in cancer correlates with disease progression, tumor load, and the severity and persistence of the associated inflammation (55, 68). Many tumors are capable of directly affecting MDSCs by secretion of proinflammatory cytokines.

VEGF, secreted by almost all tumors, induces neovascularization in the tumor microenvironment and supports tumor growth. VEGF’s effect on immune cells also takes a major role in inducing immunosuppression. Activation of STAT3 by VEGF results in induction of tolerogenic immature DCs (43). Extended treatment of mice with high-dose VEGF resulted in accumulation of Gr-1^+^ IMCs in the BM and the periphery (69). In a transgenic mouse model of spontaneously occurring melanoma, an increase in VEGF correlated with disease progression, MDSC accumulation, and immunosuppression (23). Granulocyte colony-stimulating factor (G-CSF) or granulocyte–macrophage colony-stimulating factor (GM-CSF) secreted by tumors were also shown to drive MDSC accumulation, in correlation with increased tumor progression and immunosuppression. Head and neck cancer patients with G-CSF producing tumors had increased infiltrates of suppressive CD34^+^ immature myeloid progenitors along with a reduced antitumor immune response (70). In a mouse model of spontaneous breast cancer, GM-CSF has been described as the main tumor-derived factor driving MDSC accumulation (71). GM-CSF is a potent adjuvant for vaccination since it is crucial for recruitment and activation of APCs. However, it has been shown that at a high dose, not only vaccination efficacy is lost but also MDSCs accumulate and induce immunosuppression (72). Moreover, additional soluble factors secreted by tumors were reported to affect MDSC survival and accumulation as shown for multiple myeloma cell factors that are able to induce MDSC generation through Mcl-1 upregulation (73) (Figure 1G).

### Cancer-Associated Inflammation Induces MDSC Accumulation

In addition to tumor-derived factors, the inflammatory environment of the tumor also provides signals for MDSCs. IL-1, IL-6, and TNF-α are secreted in the inflammatory settings of cancer and induce immune cell proliferation, survival, activation, and infiltration of the tumor to execute the antitumor immune response. These mediators have been shown to take a crucial part in initiation and progression of colitis-associated cancer, hepatocellular carcinoma, and chemical-induced carcinogenesis (3, 74–77). Moreover, overexpression of IL-1 was shown to induce gastric inflammation and colon cancer, mobilizing MDSCs into the inflamed colon (78). We have identified TNFα to serve as a key signal for MDSC differentiation arrest, activation, and accumulation. In particular, chronically inflamed TNF-α^−/−^ mice had reduced MDSC accumulation with low levels of ROS and NO^−^ production along with a milder T-cell dysfunction. Treatment of chronically inflamed mice with etanercept (a soluble TNF-α receptor) also diminished MDSC number and activity, recuperating T-cell function. Addition of TNF-α and GM-CSF to MDSC cultures blocked their differentiation toward DCs and macrophages compared to addition of GM-CSF alone (18). Cyclooxygenase-2 (COX-2) is overexpressed in many types of solid tumors and hematological malignancies. Prostaglandin E2 (PGE_2_) produced by this enzyme supports proliferation and survival of tumor cells by a direct interaction and serve as a key inflammatory mediator. PGE_2_ drives cell proliferation and cytokine production, contributing to overall inflammation. In the case of colon cancer, COX-2 activity has been shown to increase production of angiogenic factors and correlated with immunosuppression mediated by Tregs (79, 80). Targeting of COX-2 in 4T1 mouse mammary carcinoma resulted in reduced MDSC accumulation and relief of NK dysfunction, compared to control 4T1, when transplanted in healthy host mice (81). A PGE_2_ controlled mechanism was recently described, inducing production of CXCL12 (SDF-1) in the tumor microenvironment and expression of CXCR4 on MDSCs, mediating their recruitment in human ovarian cancer (82) (Figure 1G to A).

## Immune Monitoring and Combating MDSCs to Enhance Anticancer Treatment Efficacy

**Figure 2:**
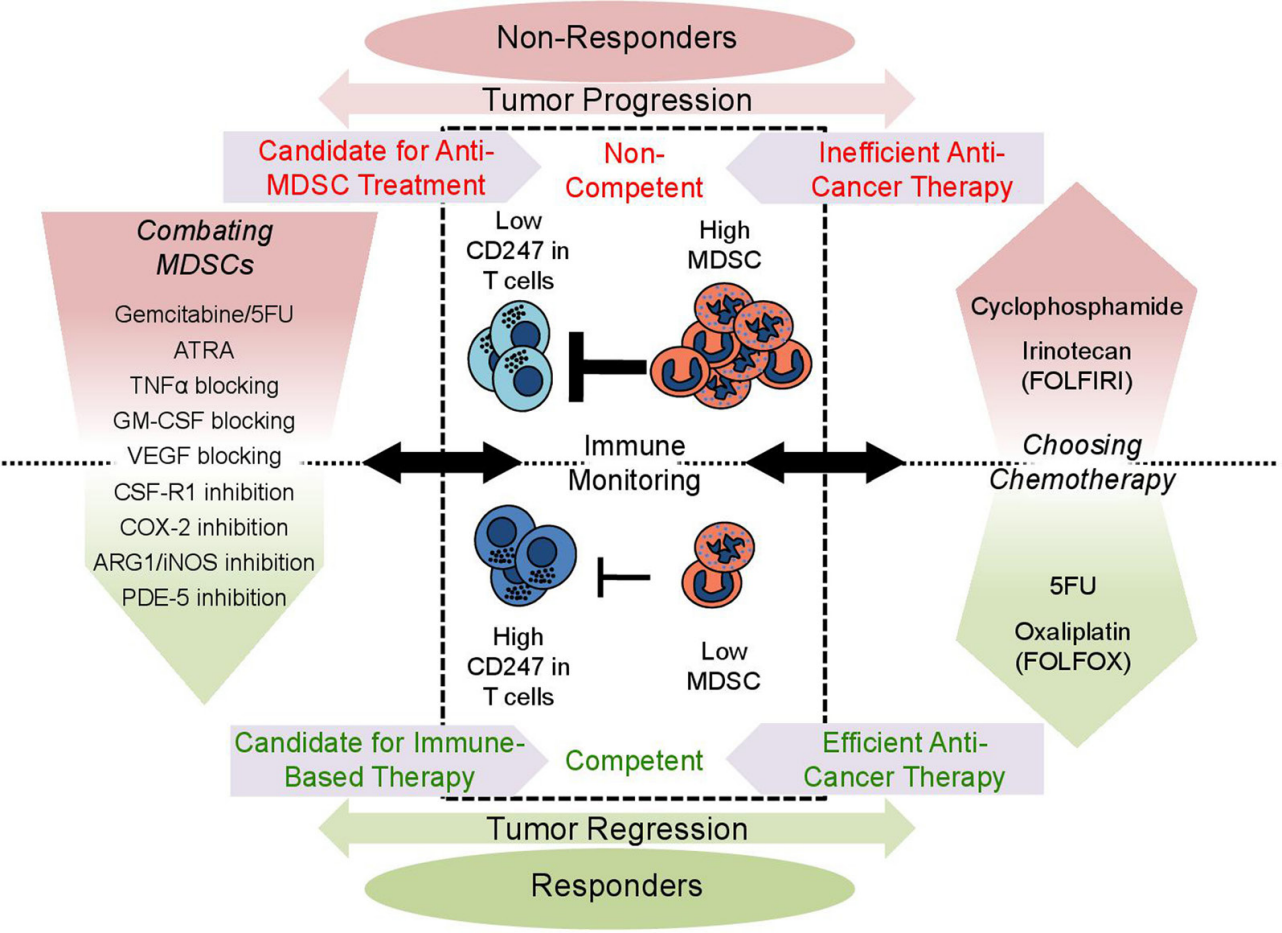
**Clinical implications of immune monitoring in cancer patients**. Monitoring of patient’s immune status in a blood sample may provide invaluable insight and help design better treatment regimens. The level of circulating MDSCs indicates how suppressive the environment is, while the expression of CD247 in circulating T-cells senses the suppression directly. Patients who have low MDSCs and high CD247 expression have a better prognosis and are more likely to respond to treatment (competent), as there are less MDSCs to support the tumor’s growth and the immune system is functional (lower part). On the other hand, high MDSC levels and low CD247 expression indicate a suppressive protumorigenic environment and immunosuppression (upper part), providing a poor prognosis (non-competent). Immune monitoring can be used when choosing a chemotherapeutic drug for treatment (right panel). Drugs such as cyclophosphamide and irinotecan induce MDSC accumulation and generate a harmful suppressive environment, while 5FU and oxaliplatin alleviate the harmful settings. Low MDSCs and high CD247 indicate immune competence making the patient a good candidate for immune-based therapy, while in suppressed and non-competent patients immune-based therapy is likely to fail. Suppressed patients may benefit from anti-MDSC treatment (left panel): MDSC elimination with chemotherapy (gemcitabine and 5FU); induction of differentiation (ATRA); blocking the signals leading to MDSC accumulation (etanercept-blocking TNF-α, anti-GM-CSF, Avastin-blocking VEGF, specific tyrosine kinase inhibitor-CSF-1R inhibitor, and Celecoxib-COX-2 inhibitor); and inhibition of MDSC activity (nitroaspirin-blocking ARG-1/iNOS and sildenafil-blocking PDE-5). Success of anti-MDSC treatment could be measured by immune monitoring, as MDSC levels drop and CD247 expression recovers, making the patient immune competent and suitable for immune-based therapy.

Immunosuppression is evident in many cases of cancer patients. Elevated MDSC levels with enhanced suppressive features inversely correlate with CD247 expression and the immune function of T- and NK-cells in the tumor site and peripheral blood, as shown for colon cancer patients (30). Immunosuppression and MDSC accumulation correlate with disease severity and provide a poor prognosis for the patients. Levels of peripheral blood MDSCs and CD247 expression serve as biomarkers capable of sensing chronic inflammation and immunosuppression. The levels of peripheral blood MDSCs and their suppressive features as measured by their production of NO and ROS can indicate the cause of the suppressive environment, while CD247 expression in T-cells is capable of sensing the resulting immunosuppression (83). Tracking these biomarkers provides new insights and understanding of the immunological effects and efficiency of anticancer treatments. Thus, following tumor parameters alongside with monitoring the immune system functional properties could increase anticancer therapy success rates and enable designing improved personalized treatments. This strategy holds for chemotherapeutic as well as immune-based treatments, as the complex dynamics within the tumor micro and macro environments involving tumor and immune cells will dictate success of both types of therapies (Figure 2).

### Chemotherapy and Immunosuppression

Chemotherapeutic drugs commonly used to treat cancer affect not only the tumor but also the immune system, having a crucial impact on antitumor responses and disease outcome. Although chemotherapies combat the tumors and lead to their regression, the effects on the tumor microenvironment and the immune system are not clearly understood. Kanterman et al. have recently shown that different chemotherapeutic agents have adverse effects on MDSC accumulation and immunosuppression (30). While treatment of colitis-induced cancer in mice with 5-fluorouracil (5FU) diminished tumor load, MDSC levels, and immunosuppression, the topoisomerase-1 inhibitor CPT-11 (irinotecan) had the opposite effect, increasing tumor load, MDSC levels, and immunosuppression. FOLFIRI and FOLFOX are two commonly used regimens in treating colon cancer patients, and until recently, their effect on the immune system had not been taken into account when choosing between them. Administration of FOLFIRI, containing irinotecan in combination with 5FU, leads to increased accumulation of MDSCs and CD247 downregulation. In contrast, the FOLFOX regimen, which contains oxaliplatin instead of irinotecan, leads to lower levels of MDSCs and recovery of CD247. Moreover, MDSCs in FOLFIRI-treated patients are more active, as indicated by increased production of NO and ROS (30). It is important to note that it is still unknown whether oxaliplatin, which is included in the FOLFOX regimen, has a direct effect on MDSCs. Another study showed that low-dose cyclophosphamide (CP) therapy that induces immunogenic tumor cell death and decreases Treg numbers had no beneficial antimelanoma effects. Instead, it increased accumulation of MDSCs, which exhibited elevated suppressive activity. Thus, melanoma therapy with low-dose CP could be efficient only when combined with the neutralization of MDSC immunosuppressive function and chronic inflammatory microenvironment (84). The cumulative data emphasize the importance of immune monitoring when choosing a chemotherapeutic treatment, as conventional measurements of chemotherapeutic effects on the tumor only are insufficient to evaluate their curative effectiveness. Moreover, as some conventional chemotherapies display significant immunotherapeutic effects, they could be used in combination with the new generation of immune-based and targeted therapies.

### Immune-Based Therapy and Immunosuppression

Activating the immune system has emerged as a promising way to treat cancer, and numerous new immune-based treatments are currently under investigation for the treatment of cancer. Recent successful phase III clinical trials of therapeutic cancer vaccines include the FDA-approved sipuleucel-T prostate cancer vaccine, melanoma peptide vaccines, and personalized lymphoma vaccines (85). Furthermore, two immune checkpoint inhibitors (anti-PD1 and anti-CTLA4) have reignited enthusiasm for the development of immunotherapeutic drugs for cancer, having demonstrated high response rates and prolonged overall survival in cancer patients (86). However, tumor-induced immunosuppression limits the potency of several standard and novel therapeutic interventions, which require a functional immune system or an environment that supports immune responses. All these therapies, as promising as they may be, risk a high probability to fail when immunosuppressive environment mediated by MDSCs is evident.

Cancer vaccines are based on presentation of tumor antigens by autologous DCs to resident T-cells in order to enhance antitumor cytotoxic activity. Dysfunctional T-cells evident in a tumor-associated immunosuppressive environment will abrogate the efficacy of such treatments. Moreover, several studies have reported difficulties to generate mature DCs *ex vivo* when the prevalence of CD14^+^ HLA-DR^−/low^ MDSCs was high in the initial samples taken from non-Hodgkin lymphoma, prostate cancer, and glioblastoma (87–90). *Ex vivo* expansion of TILs allows introduction of high numbers of tumor-reactive cytotoxic CD8^+^ T-cell clones back to the patient. Mok et al. have shown that the suppression generated by MDSCs reduces the efficacy of such treatments and that blocking CSF-1R (M-CSF receptor) reduces MDSC levels and restores the desired cytotoxic activity (91). Immune checkpoint-targeted therapy with anti-PD1 and anti-CTLA-4, which are aimed at inducing increased endogenous T-cell activity, require functional T-cells. Such treatments are expected to fail if MDSC suppressive activity is evident. Indeed, melanoma patients who had low level of MDSCs were more likely to respond to anti-CTLA-4 treatment (92, 93). Determining the patient’s immune status and immunosuppression prior to treatment can predict the likelihood to respond to such immune-based therapies.

### Combating the Tumor and Immunosuppressive Environment

The immunosuppression detected in chronic inflammation is a reversible phenomenon. Eliminating MDSCs by inducing their differentiation, inhibiting inflammatory signals that lead to their accumulation, or blocking their suppressive activity may lead to alleviation of chronic inflammation and restoration of immune functions. Various drugs and compounds, some of which are FDA approved, have been shown to target MDSCs in mouse models, attenuating chronic inflammation and inhibiting tumor growth (18, 94). Elimination of MDSCs is easily done in mice by using anti-Gr1 (RB6-8C5) monoclonal antibody (18); however, no specific markers in human MDSCs are utilized in such a manner so far. Nevertheless, gemcitabine and 5FU have been shown to specifically eliminate MDSCs by inducing apoptosis in mouse models and human patients (30, 95, 96). All-trans retinoic acid (ATRA) can potently induce MDSC differentiation into mature myeloid cells, which lose the harmful suppressive phenotype (97) and improve immune response in cancer patients (98). Blocking of various inflammatory signals prevalent in the chronically inflamed environment such as TNF-α (18), M-CSF (by blocking CSF-1R) (91), GM-CSF (99), and inhibition of COX-2 with celecoxib (100) resulted in reduced accumulation of MDSCs and decreased tumor load in mouse models. Renal cell carcinoma patients treated with bevacizumab (Avastin) to reduce VEGF-mediated angiogenesis also had reduced numbers of MDSCs in the tumors (101). Several drugs have been tested to target MDSC suppressive activity. Nitroaspirins have been shown to inhibit ARG-1 and reduce NO production and arginine consumption, thus correcting immune dysfunction and promoting tumor eradication in mice (102). Sildenafil inhibits cyclic GMP-specific phosphodiesterase type 5 (PDE-5) in MDSCs and reduces their suppressive activity, thus augmenting an antitumor response in mice *in vivo* (103). Sildenafil has also been shown to reduce Treg induction by MDSCs in a mouse model of B-cell lymphoma (45). Although various modalities capable of combating MDSCs are already FDA approved and show great promise in mouse models, there is a lack of clinical data regarding the effects of such treatments on MDSCs and chronic inflammation in cancer patients.

## Concluding Remarks

As the immune system and tumor cells intimately interact and create a complex and dynamic multifactorial micro and macro inflammatory milieu, which eventually supports tumor initiation, growth, and invasion, it is mandatory to monitor in parallel the tumor features alongside with the immune status. The cumulative data depicting the capacity of effector and regulatory immune cells to shape the environment toward supporting tumor growth highlight the critical role of chronic inflammation and associated immunosuppression, mediated by MDSCs, as a major obstacle in the success of chemo- and immune-based anticancer therapies. Only very recently, it has become clear that in order to increase the success rates of anticancer immunotherapies, a combination between monitoring the tumor responses and the patient’s immune system functionality is critical for the design of optimal treatments. Therefore, it is suggested that prior to an applied immune-based therapy, the patients must be evaluated for their immune status, using biomarkers such as CD247and MDSC levels and suppressive features. If immunosuppression is detected, it must be combated either by targeting MDSCs or by neutralizing the inflammatory environment. Upon recuperation of the immune status, immune-based therapies could then be applied. Furthermore, as described by various studies including ours, changes in the patients’ immune status also occur in response to various chemotherapies. As adverse effects of chemotherapies were obtained on the immune system functionality, some enhancing MDSC-mediated immunosuppression and others inhibiting MDSC accumulation and suppressive activity, it is mandatory to follow up chemotherapy effects not only on the tumors but also on the innate and adaptive immune system arms. Combating MDSCs and chronic inflammation, while monitoring MDSCs and CD247 in T-cells and NK cells, may provide valuable tools for choosing the correct therapeutic regiments and timing while taking into account both the cancer and immune factors. By combating MDSCs, their direct and indirect harmful effects could be alleviated on the one hand and allow reconstitution of the immune response on the other hand, thus gaining better therapy results. Moreover, monitoring the immune system functionality in the course of a given anticancer treatment could have an added value on the evaluation of tumor regression or progression as chronic inflammation and immunosuppression will decrease or increase accordingly. Therefore, by combining a monitoring system to follow in parallel changes in tumor and immune system function based on the described biomarkers, an increase in the efficacy of both immune and non-immune based anticancer therapies could be achieved and enable the design of optimal personalized treatments.

## Conflict of Interest Statement

The authors declare that the research was conducted in the absence of any commercial or financial relationships that could be construed as a potential conflict of interest.
